# Post-operative Infections After Emergent Laparotomy for Traumatic Perforated Viscera: An Assessment of Risk Factors and Antimicrobial Prophylaxis

**DOI:** 10.7759/cureus.95509

**Published:** 2025-10-27

**Authors:** Jaime B Befeler, Christian M Gill, John Culhane, Nongnooch Poowanawittayakom

**Affiliations:** 1 Department of Surgery, Saint Louis University School of Medicine, St. Louis, USA; 2 Department of Pharmacy, Sisters of Saint Mary (SSM) Health Saint Louis University Hospital, St. Louis, USA; 3 Department of Infectious Diseases, Washington University School of Medicine, St. Louis, USA

**Keywords:** antimicrobial prophylaxis, perforated hollow viscus, surgical site infection, trauma, trauma laparotomy

## Abstract

Introduction

Trauma involving perforated viscera is at high risk for surgical site infection (SSI). Standard practice is to give peri-operative prophylactic antibiotics within one hour before surgery. There is still uncertainty regarding the best specific agents and how trauma-related variables interact with prophylactic antibiotics.

Methods

This retrospective cohort study assessed patients undergoing exploratory laparotomy due to traumatic hollow visceral perforation between 1/1/2020 and 12/31/2023. The primary objective was to assess risk factors for post-operative infections. Other outcomes were death, hospital and ventilator days, and intensive care unit treatment. Significance testing for categorical and continuous data was performed with the chi-squared test and t-test, respectively. Multivariate analysis for binary and continuous outcomes was performed with logistic and linear regression, respectively.

Results

Two hundred thirty-one patients were included. SSI occurred in 26 (11%) with superficial, deep, and organ space infections documented in six, six, and 14 patients, respectively. Ertapenem was the most common antimicrobial prophylaxis (168, 73%). Antimicrobial prophylaxis without expected activity against anaerobes was given in 46 patients (20%). No antimicrobial prophylaxis was administered in 10 patients (4%). Antimicrobials were administered within 60 minutes prior to the procedure in 150 patients (65%).

Patients who received peri-operative antibiotic prophylaxis with any agent had six SSIs (2.9%) versus four SSIs (15.4%) for patients who received no antibiotic prophylaxis of any kind (p=0.017). Patients who received ertapenem prophylaxis had one trauma-related SSI (0.6%) versus four SSIs (6.2%) for patients who received antibiotic prophylaxis with any other agent (p=0.008). On multivariate analysis, the adjusted odds ratio for infection associated with ertapenem prophylaxis versus any other agent was protective at 0.05 (p=0.01). Hospital length of stay (LOS) was 20.3 days for patients who received post-operative antibiotics versus 12.1 days for those who did not receive post-operative antibiotics (p<0.01).

Patients who received massive transfusion protocol (MTP) had eight SSIs (22.2%) versus 17 SSIs (8.8%) for those who did not receive MTP (p=0.02). Patients who received post-operative vasopressors had nine SSIs (22%) versus 17 SSIs (9%) for those who did not receive post-operative vasopressors (p=0.02). Patients whose abdominal fascia was left open at the end of the index operation had 21 SSIs (16.4%) versus five SSIs (4.9%) for those whose fascia was closed immediately (p=0.01).

Conclusion

Lack of antibiotic prophylaxis, MTP, post-operative vasopressors, and open abdomen predict SSI. Ertapenem was associated with a lower traumatic wound infection rate, but not SSI. Other specific antibiotic agents and timing intervals were not significant. Variables that are surrogates for trauma severity are more accurate predictors of SSI than specific antibiotic agents and timing.

## Introduction

Surgical site infections (SSIs) occur in roughly 2% of all surgical procedures and account for approximately 20% of all healthcare-associated infections, making them one of the most common nosocomial infections among patients undergoing surgery [1-3]. SSIs present an important burden for both patients and healthcare systems, often leading to prolonged hospital stays and increased healthcare costs [4]. Surgery involving the gastrointestinal (GI) tract poses an increased risk for SSI due to the proximity of bacterial flora that colonize the area [5-7]. These risks are further heightened in the setting of emergency procedures (e.g., exploratory laparotomy) after trauma as spillage from the GI tract may contaminate the peritoneal cavity prior to surgery [5,8,9].

Although the introduction of quality bundles such as Enhanced Recovery After Surgery (ERAS) protocols has decreased complications in elective colorectal procedures, such measures are challenging in the emergent setting due to the lack of time for preparatory interventions, along with the acute nature of the procedure, and the presentation with physiologic abnormalities [10,11]. Although there are limited randomized controlled trial data, peri-operative antimicrobial prophylaxis has remained a cornerstone of preventing SSI in the setting of acute hollow visceral trauma [6,9,12-14]. No agent has been adequately shown to improve outcomes over another, but typically agents with activity against aerobic gram-positive, aerobic gram-negative, and anaerobic pathogens are recommended and associated with positive clinical outcomes [12-15]. Recent quality improvement data show that a peri-operative bundle including ertapenem for peri-operative prophylaxis showed a decrease in SSI [6,16]. This data led to updates in national guidelines; however, confirmation of benefit is still pending. 

There remains uncertainty regarding the best specific antimicrobial agent in trauma and how trauma-related variables interact with prophylactic antibiotics for preventing SSI. To address this, we conducted a retrospective cohort study aimed at examining the incidence, characteristics, and risk factors of SSI in patients with full-thickness hollow visceral trauma in association with prophylactic antibiotics. The primary objective was to assess the risk factors for post-operative infections in patients who underwent exploratory laparotomy due to traumatic visceral perforation. Secondary objectives were to evaluate the post-operative outcomes including hospital length of stay (LOS), days on mechanical ventilation, and length of intensive care unit (ICU) stay. Understanding how trauma-related variables interact with prophylactic antibiotics is crucial for developing effective preventive strategies and improving patient outcomes.

## Materials and methods

Study design and population

This retrospective cohort study assessed patients at Saint Louis University Hospital, St. Louis, USA, an urban Level 1 trauma center and academic institution, undergoing exploratory laparotomy due to traumatic visceral perforation between 1/1/2020 and 12/31/2023. Patients were identified through the Saint Louis University Hospital Trauma Registry and screened for inclusion. All patients who underwent emergent laparotomy due to trauma were eligible for inclusion. Exclusion criteria included lack of laparotomy and death during resuscitation or during the index procedure. The study was reviewed by Saint Louis University Institutional Review Board (protocol number: 33783) and was deemed exempt under category 4.

Data obtained from the existing registry include the baseline characteristics of age, sex, body mass index (BMI), injury type (either blunt injury or penetrating), Injury Severity Score (ISS), and Trauma and Injury Severity Score (TRISS).

Outcomes include mortality, ICU days, ventilator days, LOS, and post-operative infections. Trauma-related data manually abstracted from the electronic health record include delayed fascial closure (open abdomen), presence of a colon or rectal injury, need for post-operative vasopressors, and massive transfusion protocol (MTP). Details about antimicrobial prophylaxis were collected including the agent(s) used, timing, dose, and occurrence of repeated dosing. 

Outcomes

Clinical outcomes were assessed including the occurrence of post-operative infections within 30 days. Infections were categorized per the National Healthcare Safety Network (NHSN) criteria as superficial, deep, or organ space infections [1]. 

The primary objective was to assess risk factors for SSIs. Secondary outcomes included hospital LOS, days on mechanical ventilation, and length of ICU stay. During the study period, ertapenem 1 g IV administered within 60 minutes of the procedure start was the recommended surgical infection prophylaxis (SIP) for trauma-related exploratory laparotomy. Since no one agent or combination is the accepted standard of care for SIP in this setting, SIP was deemed appropriate if agents had expected activity against gram-positive, gram-negative, and anaerobic bacteria per the Eastern Association for the Surgery of Trauma (EAST) guidelines that were administered within 60 minutes of the procedure (e.g., ertapenem, cefazolin plus metronidazole, piperacillin/tazobactam, etc.) [9,15].

Statistical analysis 

Univariate Analysis

Four antibiotic-related exposures were chosen as independent predictor variables: peri-operative prophylaxis with ertapenem versus other agents, peri-operative prophylactic antibiotics given within the correct time frame, antibiotics redosed intra-operatively, and whether the patient received antibiotics after surgery. Trauma-related independent variables included massive transfusion, colorectal injury, post-operative vasopressors, and open abdomen after the index procedure.

Continuous outcome variables of ICU days, hospital days, and ventilator days were compared between antibiotic and trauma-related exposure groups. Binary outcomes compared between these groups are death and the infection-related outcome of any trauma-related soft tissue infection. These infections include superficial SSI, deep SSI, organ space SSI, any SSI, traumatic wound infection, and intraabdominal abscess. Traumatic wound infection refers to the superficial component of wounds resulting directly from trauma, generally bullet holes and knife wounds. The t-test was used for significance testing for continuous outcomes. The chi-squared test was used for categorical outcomes.

Multivariate Analysis

Independent variables were controlled for age, gender, mechanism (i.e., blunt or penetrating trauma), ISS, and TRISS. Multivariate logistic regression was used for binary outcomes, and multivariate linear regression was used for continuous outcomes. A p-value of <0.05 was considered significant. All statistics were performed with the R Project Version 4.3.0 (R Foundation for Statistical Computing, Vienna, Austria).

## Results

A total of 304 patients presenting to the Saint Louis University Hospital Emergency Department with penetrating colorectal trauma between 1/1/2020 and 12/31/2023 were screened for inclusion. Patients of ages 18-110 years old, any gender, and any ethnic background were included. Patients were excluded if they died during pre-operative resuscitation, upon arrival, or intra-operatively. Patients who did not undergo an exploratory laparotomy during the index admission were also excluded. 

A total of 231 patients were included in the analysis. SSI occurred in 26 patients (11%). Superficial, deep, and organ space infections accounted for in six, six, and 14 patients, respectively. 

Ertapenem was the most common antimicrobial prophylaxis, used for 168 patients (73%). Antimicrobial prophylaxis without expected activity against anaerobes was administered for 46 patients (20%). No antimicrobial prophylaxis was administered for 10 patients (4%). Antimicrobials were administered within 60 minutes prior to the procedure in 150 patients (65%). Post-operative infection was more common among patients who received no antibiotic prophylaxis (four, 15.4%) versus those who received prophylaxis with any agent (six, 2.9%) (p=0.02).

Tables 1-4 present the baseline characteristics stratified by exposure group. Four antibiotic-related exposures are displayed: peri-operative antibiotic prophylaxis with ertapenem versus another agent, antibiotic prophylaxis given within the correct time frame (<1 hour prior to incision), antibiotics redosed intra-operatively, and whether the patient received antibiotics after surgery. Trauma studies traditionally are adjusted for age, gender, mechanism (blunt versus penetrating), an anatomic severity score (ISS), and a physiologic severity score (TRISS). These variables were chosen as the most relevant baseline characteristics. Further breakdown of outcomes associated with antibiotic use and indices of trauma severity is displayed in Table 2 and Table 3.

**Table 1:** Baseline characteristic with ertapenem vs other antibiotics The chi-squared test (^χ2^) and t-test (^T^) were used. SMD: standardized mean difference; SD: standard deviation; BMI: body mass index; ISS: Injury Severity Score; TRISS: Trauma and Injury Severity Score

Baseline characteristic	Antibiotic other than ertapenem	Ertapenem	p	SMD	Test statistic
n	64	167	-	-	-
Age (mean±SD)	36.61±14.63	34.41±14.67	0.309	0.15	1.02^T^
Female gender (%)	9 (14.1)	28 (16.8)	0.763	0.075	0.09^χ2^
Penetrating injury (%)	41 (64.1)	140 (83.8)	0.002	0.462	9.5^χ2^
BMI (mean±SD)	28.30±7.50	27.57±6.86	0.48	0.102	0.68^T^
ISS (mean±SD)	22.33±14.76	21.01±11.36	0.469	0.1	0.65^T^
TRISS (mean±SD)	0.81±0.28	0.89±0.21	0.024	0.313	-2.0^T^​​​​​​​

**Table 2:** Baseline characteristic with timing of antibiotics The chi-squared test (^χ2^) and t-test (^T^) were used. The total does not equal 231 due to incomplete records. Missing data is treated by listwise deletion. SMD: standardized mean difference; SD: standard deviation; BMI: body mass index; ISS: Injury Severity Score; TRISS: Trauma and Injury Severity Score

Baseline characteristic	Antibiotics >1 hour	Antibiotics <1 hour	p	SMD	Test statistic
n	80	150	-	-	-
Age (mean±SD)	33.99±12.67	35.55±15.68	0.443	0.11	-0.82^T^
Female gender (%)	16 (20)	20 (13.3)	0.256	0.18	1.29^χ2^
Penetrating injury (%)	58 (72.5)	122 (81.3)	0.168	0.211	1.90^χ2^
BMI (mean±SD)	28.01±6.80	27.60±7.17	0.673	0.059	0.43^T^
ISS (mean±SD)	21.75±13.55	21.20±11.78	0.749	0.043	0.31^T^
TRISS (mean±SD)	0.84±0.26	0.89±0.22	0.157	0.192	-1.35^T^

**Table 3:** Baseline characteristic with intra-operative redosing The chi-squared test (^χ2^) and t-test (^T^) were used. The total does not equal 231 due to incomplete records. Missing data is treated by listwise deletion. SMD: standardized mean difference; SD: standard deviation; BMI: body mass index; ISS: Injury Severity Score; TRISS: Trauma and Injury Severity Score

Baseline characteristic	No intraoperative redose	Intraoperative redose	p	SMD	Test statistic
n	193	35	-	-	-
Age (mean±SD)	34.93±14.47	34.71±15.99	0.937	0.014	0.07^T^
Female gender (%)	30 (15.5)	7 (20)	0.683	0.117	0.17^χ2^
Penetrating injury (%)	154 (79.8)	24 (68.6)	0.21	0.259	0.65^χ2^
BMI (mean±SD)	27.44±6.78	29.03±8.06	0.219	0.213	-1.09^T^
ISS (mean±SD)	21.15±12.20	22.69±13.54	0.5	0.12	-0.63^T^
TRISS (mean±SD)	0.89±0.22	0.79±0.29	0.019	0.394	1.58^T^

**Table 4:** Baseline characteristic with antibiotics after surgery The chi-squared test (^χ2^) and t-test (^T^) were used. SMD: standardized mean difference; SD: standard deviation; BMI: body mass index; ISS: Injury Severity Score; TRISS: Trauma and Injury Severity Score

Baseline characteristic	No antibiotics after surgery	Antibiotics after surgery	p	SMD	Test statistic
n	79	152	-	-	-
Age (mean±SD)	37.04±16.80	33.97±13.36	0.132	0.202	1.41^T^
Female gender (%)	16 (20.3)	21 (13.8)	0.282	0.172	1.16^χ2^
Penetrating injury (%)	59 (74.7)	122 (80.3)	0.419	0.134	0.65^χ2^
BMI (mean±SD)	27.07±6.83	28.14±7.13	0.274	0.154	-1.11^T^
ISS (mean±SD)	17.78±10.99	23.24±12.68	0.001	0.46	-3.39^T^
TRISS (mean±SD)	0.91±0.17	0.85±0.26	0.066	0.274	2.09^T^

Tables 5-8 present the selected clinical and infectious outcomes associated with the antibiotic exposure groups. We include the continuous outcome variables of ICU days, hospital days, and ventilator days. The only significant difference for the continuous variables is a hospital stay of 20.3 days for patients who received antibiotics after surgery versus 12.1 days for those who did not (p<0.001). The only significant antibiotic-related association among the binary outcomes is prophylactic ertapenem with traumatic wound infection. Four patients (6.2%) without ertapenem suffered infection versus one (0.6%) with ertapenem (p=0.008).

**Table 5:** Outcomes with ertapenem vs other antibiotics The chi-squared test (^χ2^) and t-test (^T^) were used. ICU: intensive care unit; SSI: surgical site infection; SD: standard deviation

	Antibiotic other than ertapenem	Ertapenem	p	Test statistic
n	64	167	-	-
Hospital days (mean±SD)	17.9±16.5	17.3±16.3	0.83	0.21^T^
ICU days (mean±SD)	9.8±7.8	8.9±9.2	0.55	0.61^T^
Ventilator days (mean±SD)	7.2±6.6	6.9±8.6	0.8	0.25^T^
Death (%)	3 (4.7)	6 (3.6)	0.7	0.15^χ2^
Any infection (%)	9 (14.1)	17 (10.2)	0.4	0.70^χ2^
Superficial SSI (%)	0 (0)	1 (0.6)	0.53	0.38^χ2^
Deep SSI (%)	2 (3.1)	2 (1.2)	0.31	1.01^χ2^
Organ space SSI (%)	0 (0)	2 (1.2)	0.38	0.77^χ2^
Any SSI (%)	2 (3.1)	5 (3)	0.96	0.003^χ2^
Traumatic wound infection (%)	4 (6.2)	1 (0.6)	0.008	6.98^χ2^
Intraabdominal abscess (%)	3 (4.7)	10 (6)	0.7	0.15^χ2^

**Table 6:** Outcomes with timing of antibiotics The chi-squared test (^χ2^) and t-test (^T^) were used. The total does not equal 231 due to incomplete records. Missing data is treated by listwise deletion. ICU: intensive care unit; SSI: surgical site infection; SD: standard deviation

	Antibiotics >1 hour	Antibiotics <1 hour	p	Test statistic
n	80	150	-	-
Hospital days (mean±SD)	19±17.6	16.7±15.6	0.31	1.02^T^
ICU days (mean±SD)	9.3±7.7	9±9.6	0.86	0.18^T^
Ventilator days (mean±SD)	6.7±6.2	7.1±9.1	0.76	-0.30^T^
Death (%)	3 (3.7)	6 (4)	0.93	0.009^χ2^
Any infection (%)	11 (13.8)	14 (9.3)	0.31	1.05^χ2^
Superficial SSI (%)	0 (0)	1 (0.7)	0.31	0.54^χ2^
Deep SSI (%)	3 (3.7)	1 (0.7)	0.09	2.90^χ2^
Organ space SSI (%)	1 (1.2)	1 (0.7)	0.65	0.21^χ2^
Any SSI (%)	4 (5)	3 (2)	0.21	1.59^χ2^
Traumatic wound infection (%)	3 (3.7)	2 (1.3)	0.23	1.43^χ2^
Intraabdominal abscess (%)	5 (6.2)	8 (5.3)	0.77	0.08^χ2^

**Table 7:** Outcomes with intra-operative redosing The chi-squared test (^χ2^) and t-test (^T^) were used. The total does not equal 231 due to incomplete records. Missing data is treated by listwise deletion. ICU: intensive care unit; SSI: surgical site infection; SD: standard deviation

	No intra-operative redosing	Intra-operative redosing	p	Test statistic
n	193	35	-	-
Hospital days (mean±SD)	17.5±16.5	17.7±15.3	0.94	-0.07^T^
ICU days (mean±SD)	9.2±9.3	8.7±7.0	0.76	0.31^T^
Ventilator days (mean±SD)	7.1±8.6	6.1±4.7	0.57	0.58^T^
Death (%)	7 (3.6)	2 (5.7)	0.56	0.34^χ2^
Any infection (%)	22 (11.4)	3 (8.6)	0.62	0.24^χ2^
Superficial SSI (%)	1 (0.5)	0 (0)	0.67	0.18^χ2^
Deep SSI (%)	4 (2.1)	0 (0)	0.39	0.74^χ2^
Organ space SSI (%)	2 (1)	0 (0)	0.55	0.37^χ2^
Any SSI (%)	7 (3.6)	0 (0)	0.25	1.31^χ2^
Traumatic wound infection (%)	3 (1.6)	1 (2.9)	0.59	0.29^χ2^
Intraabdominal abscess (%)	11 (5.7)	2 (5.7)	0.99	<0.01^χ2^

**Table 8:** Outcomes with antibiotics after surgery The chi-squared test (^χ2^) and t-test (^T^) were used. ICU: intensive care unit; SSI: surgical site infection; SD: standard deviation

	No antibiotics after surgery	Antibiotics after surgery	p	Test statistic
n	79	152	-	-
Hospital days (mean±SD)	12.1±8.1	20.3±18.6	<0.001	-12.54^T^
ICU days (mean±SD)	7.3±6.4	9.9±9.7	0.08	-1.76^T^
Ventilator days (mean±SD)	5.7±6.2	7.5±8.7	0.22	-4.63^T^
Death (%)	5 (6.3)	4 (2.6)	0.17	1.90^χ2^
Any infection (%)	8 (10.1)	18 (11.8)	0.7	0.15^χ2^
Superficial SSI (%)	0 (0)	1 (0.7)	0.47	0.52^χ2^
Deep SSI (%)	2 (2.5)	2 (1.3)	0.5	0.45^χ2^
Organ space SSI (%)	1 (1.3)	1 (0.7)	0.64	0.22^χ2^
Any SSI (%)	3 (3.8)	4 (2.6)	0.62	0.24^χ2^
Traumatic wound infection (%)	2 (2.5)	3 (2)	0.78	0.08^χ2^
Intraabdominal abscess (%)	4 (5.1)	9 (5.9)	0.79	0.07^χ2^

Tables 9-12 present a univariate analysis of infection-related outcomes associated with trauma-related variables selected as indices of trauma severity and increased risk of infectious complication.

**Table 9:** Trauma-related predictors with colorectal injury The chi-squared test (^χ2^) was used. SSI: surgical site infection

	No colorectal injury	Colorectal injury	p	Test statistic (^χ2^)
n	62	169	-	-
Death (%)	3 (4.6)	6 (3.6)	0.72	0.12
Any infection (%)	4 (6.2)	22 (13.3)	0.12	2.36
Superficial SSI (%)	0 (0)	1 (0.6)	0.53	0.39
Deep SSI (%)	1 (1.5)	3 (1.8)	0.89	0.02
Organ space SSI (%)	0 (0)	2 (1.2)	0.37	0.79
Any SSI (%)	1 (1.5)	6 (3.6)	0.41	0.69
Traumatic wound infection (%)	0 (0)	5 (3)	0.16	2.00
Intraabdominal abscess (%)	3 (4.6)	10 (6)	0.68	0.17

**Table 10:** Trauma-related predictors with MTP The chi-squared test (^χ2^) was used. MTP: massive transfusion protocol; SSI: surgical site infection

	No MTP	MTP	p	Test statistic (^χ2^)
n	201	30	-	-
Death (%)	8 (4.1)	1 (2.8)	0.7	0.15
Any infection (%)	17 (8.8)	8 (22.2)	0.02	5.68
Superficial SSI (%)	1 (0.5)	0 (0)	0.67	0.19
Deep SSI (%)	3 (1.5)	1 (2.8)	0.6	0.27
Organ space SSI (%)	2 (1)	0 (0)	0.54	0.37
Any SSI (%)	6 (3.1)	1 (2.8)	0.92	0.01
Traumatic wound infection (%)	1 (1.5)	3 (8.3)	<0.001	10.86
Intraabdominal abscess (%)	10 (5.2)	3 (8.3)	0.45	0.58

**Table 11:** Trauma-related predictors with post-operative vasopressors The chi-squared test (^χ2^) was used. SSI: surgical site infection

	No post-operative vasopressors	Post-operative vasopressors	p	Test statistic (^χ2^)
n	189	41	-	-
Death (%)	7 (3.7)	2 (4.9)	0.73	0.12
Any infection (%)	17 (9)	9 (22)	0.02	5.64
Superficial SSI (%)	0 (0)	1 (2.4)	0.4	0.71
Deep SSI (%)	4 (2.1)	0 (0)	0.35	0.88
Organ space SSI (%)	1 (0.5)	1 (2.4)	0.23	1.43
Any SSI (%)	5 (2.6)	2 (4.9)	0.45	0.57
Traumatic wound infection (%)	3 (1.6)	2 (4.9)	0.19	1.72
Intraabdominal abscess (%)	9 (4.8)	4 (9.8)	0.21	1.58

**Table 12:** Trauma-related predictors with an open abdomen The chi-squared test (^χ2^) was used. SSI: surgical site infection

	No open abdomen	Open abdomen	p	Test statistic (^χ2^)
n	103	128	-	-
Death (%)	6 (5.8)	3 (2.3)	0.17	1.85
Any infection (%)	5 (4.9)	21 (16.4)	0.006	7.62
Superficial SSI (%)	0 (0)	1 (0.8)	0.37	0.81
Deep SSI (%)	1 (1)	3 (2.3)	0.43	0.63
Organ space SSI (%)	0 (0)	2 (1.6)	0.2	1.62
Any SSI (%)	1 (1)	6 (4.7)	0.1	2.69
Traumatic wound infection (%)	2 (1.9)	3 (2.3)	0.83	0.04
Intraabdominal abscess (%)	2 (1.9)	11 (8.6)	0.03	4.76

For trauma-related variables, the following associations were significant. Seventeen patients (8.8%) without MTP got any infection versus eight (22.2%) who received MTP (p=0.02). One patient (0.5%) without MTP versus three patients (8.3%) with MTP had a wound infection (p <0.001). Five patients (4.9%) without an open abdomen following the initial surgery suffered any infection versus 21 (16.4%) of those with an open abdomen (p=0.006). Two patients (1.9%) without an open abdomen had an intraabdominal abscess versus 11 (8.6%) for those with an open abdomen (p=0.03). Seventeen patients (9%) not requiring post-operative vasopressors suffered any infection versus nine (22%) for those with post-operative vasopressors (p=0.02).

The multivariate analyses included the multivariate logistic regressions of death and the infection-related outcomes with antibiotic-related predictors as presented in Table 13. Only one antibiotic-related variable was an independent predictor of an infection-related outcome. Prophylactic ertapenem was an independent predictor of less traumatic wound infection. The odds ratio of traumatic wound infection was 0.05 for ertapenem prophylaxis versus no ertapenem prophylaxis (p=0.01).

**Table 13:** Multivariate logistic regression of traumatic wound infection with prophylactic ertapenem and standard demographic/trauma covariates For the dichotomous variables of ertapenem prophylaxis, gender, and injury type, the reference category is 0, representing no ertapenem prophylaxis, male gender, and blunt injury type. ISS: Injury Severity Score; TRISS: Trauma and Injury Severity Score; 95% CI: 95% confidence interval

Variable	Estimate	Standard error	z value	Odds ratio (95% CI)	p
(Intercept)	-22.34	2165	-0.01	<0.001	0.9918
Ertapenem prophylaxis	-2.99	1.18	-2.53	0.0502 (0.0024-0.38)	0.0114
Age	-0.00918	0.0377	-0.24	0.991 (0.91-1.06)	0.8078
Gender	1.13	1.27	0.89	3.1 (0.135-33.1)	0.3745
TRISS	0.682	2.36	0.29	1.98 (0.034-1728)	0.7729
ISS	0.0597	0.0461	1.3	1.06 (0.97-1.16)	0.1954
Injury type	18.5	2165	0.01	>1000	0.9932

Multivariate linear regressions for continuous outcomes showed one independent association as presented in Table 14. Receiving antibiotics after surgery was an independent predictor of increased LOS. Those who got antibiotics after surgery stayed an average of 6.25 days longer (p=0.004). 

**Table 14:** Linear regression of length of stay in days with administration of antibiotics after surgery and standard demographic/trauma covariates For the dichotomous variables of ertapenem prophylaxis, gender, and injury type, the reference category is 0, representing no ertapenem prophylaxis, male gender, and blunt injury type. Abx: antibiotics; ISS: Injury Severity Score; TRISS: Trauma and Injury Severity Score; 95% CI: 95% confidence interval

Variable	Estimate (95% CI)	Standard error	t value	p
(Intercept)	13.02449 (-1.22, 27.27)	7.23031	1.801	0.07301
Abx after surgery	6.25345 (2.03, 10.48)	2.14396	2.917	0.0039
Age	0.11538 (-0.02, 0.25)	0.06948	1.661	0.09821
Gender	-3.97424 (-9.41, 1.46)	2.75836	-1.441	0.15106
TRISS	-8.62254 (-19.03, 1.78)	5.27899	-1.633	0.10381
ISS	0.31492 (0.12, 0.51)	0.1013	3.109	0.00212
Injury type	-2.84427 (-8.23, 2.54)	2.73236	-1.041	0.29903

## Discussion

Hollow viscus injury after trauma is a surgical emergency necessitating prompt intervention. Post-surgical infection after exploratory laparotomy remains a clinical challenge. Observational studies assessing the current practices and characteristics of patients who developed SSIs after emergent laparotomy are crucial to target continuous quality improvement. 

Peri-operative antimicrobial prophylaxis remains one of the most important interventions to prevent post-operative infections. Although no randomized controlled trials have demonstrated the benefit of antimicrobial prophylaxis compared with placebo, since this practice became standard in the early 21st century, sepsis-related mortality post-laparotomy has decreased [17]. Contemporary guidelines support the administration of antimicrobials targeting gram-positive aerobes, gram-negative aerobes, and anaerobic bacteria [6,7,9]. The Surgical Care Improvement Project (SCIP) recommends several peri-operative measures to help reduce the number of preventable surgical complications across the country. Their goals are to improve surgical care processes, standardize procedures, and reduce SSI. They recommend the use of prophylactic antibiotics within one hour before surgical incisions, selecting appropriate antibiotics and then discontinuing them within 24 hours post-surgery [18]. Evidence from a meta-analysis looking at the timing of pre-operative antibiotic prophylaxis in 54,552 patients demonstrated that the administration of antibiotics 60-0 minutes prior to incisions was associated with lower SSI rates compared to administering antibiotic prophylaxis 120-60 minutes before surgery or after initial surgical incision [19]. Although compelling data suggest peri-operative antimicrobials are integral in preventing post-operative infections after trauma laparotomy, the optimal choice of antimicrobial agent is less well established [15]. 

Our results show only one significant association with ertapenem and infectious outcome: the protective association of ertapenem with traumatic wound infection. The magnitude of the benefit along with the lack of protective association with other outcome variables leads us to question the plausibility of this finding. It is most likely a statistical anomaly due to low numbers. Prior research has promoted the importance of a standardized antimicrobial prophylaxis protocol, but the data are mixed on which specific agent is best [12-14]. A randomized study of patients undergoing elective colorectal surgery by Itani et al. in 2006 showed that ertapenem is more effective than cefotetan in the prevention of SSI. In the ertapenem group, 338 (17.1%) had infection versus 334 (26.2%) in the cefotetan group (absolute difference: -9.1; 95% CI: -14.4 to -3.7) [20]. The advantage of ertapenem in this study is attributed to its long half-life and spectrum of activity including anaerobic organisms. However, observational data that include trauma-related laparotomy are conflicting. A single-center, pre-post study showed that ertapenem as the standard prophylactic agent for trauma laparotomy was associated with lower rates of SSI [16]. Conversely, a large multicenter observational study of both elective and emergent laparotomies found a higher risk of SSI in ertapenem-treated patients compared with other peri-operative antimicrobials (e.g., first- and second-generation cephalosporins, fluoroquinolones). In the propensity-weighted analysis, ertapenem prophylaxis was associated with higher odds of SSI (1.56; 95% CI: 1.08-2.26; p=0.02) [21]. Although most patients in the present study received ertapenem as peri-operative prophylaxis, receipt of ertapenem was associated with less traumatic wound infection but not total SSI. Concordant with previous literature, the lack of any peri-operative antimicrobial prophylaxis was associated with increased SSI. This confirms the importance of prophylaxis, but there remains equipoise surrounding the superiority of one agent over others. 

Our results with respect to antimicrobial use must be taken in the context of numerous other factors likely contributing to SSI, most notably the extent of injury and prompt surgical infectious source control. Rates of SSI in trauma surgery have been attributed to trauma-related variables. A retrospective study of the Trauma Quality Improvement Database of 41,034 patients by Durbin et al. found that in abdominal trauma patients who underwent exploratory laparotomy, the incidence of SSI increases with increased BMI, increased age, location of injury, blood transfusion needs, and increasing number of hollow viscus injuries. The conclusion was that the presence of these injuries is a major risk factor for SSI due to the bacterial load of the GI tract [22]. Additionally, other factors include timing of surgical intervention. Wei et al. demonstrated that surgical treatment within 22 hours of injury significantly reduces mortality and sepsis rates in patients with blunt hollow viscus injuries [23]. Several studies have also found an association between high blood product transfusion requirements and SSI in trauma patients [24]. One potential explanation for this finding is that patients with a higher severity of initial injury are predisposed to both higher rates of SSI and bleeding complications. Lower plasma concentration of antimicrobial agents has been offered as another explanation for the association of infection with blood transfusion [25]. The high blood loss and resuscitation may predispose to sub-therapeutic antimicrobial exposure which prompts clinical guidelines to recommend the redosing of antimicrobials after >10 units of blood products are administered [9,24]. If antibiotic redosing is missed, this may lead to antimicrobial underexposure and an increased risk of post-operative SSI. Redosing may also be a surrogate for longer procedures expected with more severe injuries. Our results did not show an association of infection with intra-operative redosing, perhaps because better antimicrobial efficacy was opposed by an association with more severe injury. More data are needed to assess the association of massive transfusion and SSIs including the interplay of antimicrobial pharmacokinetics/pharmacodynamics with trauma severity. 

Our study confirms that any antimicrobial prophylaxis is more effective than none, but beyond that, the details of antibiotic administration had little effect on outcome. We found that variables that are surrogates for trauma severity such as MTP, post-operative pressors, and open abdomen contribute more to SSI rates. This raises the question of whether SSI in trauma should be a quality metric for hospital reimbursement. Our results suggest that SSI rates in trauma are more strongly associated with trauma severity than the details of antibiotic administration. As a result, centers with higher rates of severe trauma and more complex trauma operations may be more at risk for financial penalty [11]. With that said, the clinically important rate of SSI in the present study and others highlight the need for continuing quality improvement in the care of high-risk trauma patients [13]. In addition to refining the best choice of prophylactic antimicrobials, interventions that improve surgical source control and safety are logical targets for improvement to reduce SSI [26].

Limitations 

As a single-center study, ours does not have the statistical power of a large registry. Type 2 error is a possible explanation where we see negative results. The protective association of ertapenem prophylaxis with wound infection on multivariate analysis should be interpreted with caution. It seems implausible that the odds of wound infection are 20-fold lower with ertapenem prophylaxis when we saw no significant associations with total infection. This finding is intriguing but requires confirmation with further study.

As a retrospective chart review, this study is more hypothesis-generating than practice-changing. A clinician would not likely make clinical decisions based on the association of ertapenem with traumatic wound infection, but an investigator might follow it up with further study. This is the value of reporting the result.

## Conclusions

SSI is a clinically important source of morbidity after exploratory laparotomy for traumatic perforated viscera. Lack of prophylactic antibiotics but not timing of administration predicted SSI. There is a weak but statistically significant protective association with ertapenem prophylaxis. Trauma-related variables showed more numerous and stronger associations with infectious outcome than antibiotic-related variables. These include MTP, open abdomen, and need for post-operative vasopressors. The persistence of SSI in trauma shows a need for continuing quality improvement. Based on our results, the severity of trauma rather than the details of prophylactic antibiotic use is the most important factor in SSI rate. While we should continue to strive to improve trauma care, trauma severity is not a risk factor modifiable by clinical practice. This has implications for the use of SSI as an index of quality of care.

## References

[1] (2024). Surgical Site Infection Event (SSI). https://www.cdc.gov/nhsn/pdfs/pscmanual/9pscssicurrent.pdf.

[2] Berríos-Torres SI, Umscheid CA, Bratzler DW (2017). Centers for Disease Control and Prevention guideline for the prevention of surgical site infection, 2017. JAMA Surg.

[3] Stevens DL, Bisno AL, Chambers HF (2014). Practice guidelines for the diagnosis and management of skin and soft tissue infections: 2014 update by the Infectious Diseases Society of America. Clin Infect Dis.

[4] De Simone B, Sartelli M, Coccolini F (2020). Intraoperative surgical site infection control and prevention: a position paper and future addendum to WSES intra-abdominal infections guidelines. World J Emerg Surg.

[5] Ruiz-Tovar J, Boermeester MA, Bordeianou L (2022). Delphi consensus on intraoperative technical/surgical aspects to prevent surgical site infection after colorectal surgery. J Am Coll Surg.

[6] Bratzler DW, Dellinger EP, Olsen KM (2013). Clinical practice guidelines for antimicrobial prophylaxis in surgery. Surg Infect (Larchmt).

[7] Huston JM, Barie PS, Dellinger EP (2024). The Surgical Infection Society guidelines on the management of intra-abdominal infection: 2024 Update. Surg Infect (Larchmt).

[8] Biffl WL, Leppaniemi A (2015). Management guidelines for penetrating abdominal trauma. World J Surg.

[9] Goldberg SR, Anand RJ, Como JJ (2012). Prophylactic antibiotic use in penetrating abdominal trauma: an Eastern Association for the Surgery of Trauma practice management guideline. J Trauma Acute Care Surg.

[10] Cavallaro P, Bordeianou L (2019). Implementation of an ERAS pathway in colorectal surgery. Clin Colon Rectal Surg.

[11] Fornek ML, Ata S, Jimenez E (2024). Reportable infections following colon surgery in a large public healthcare system in New York City: the consequences of being a level 1 trauma center. Infect Control Hosp Epidemiol.

[12] Delgado G Jr, Barletta JF, Kanji S, Tyburski JG, Wilson RF, Devlin JW (2002). Characteristics of prophylactic antibiotic strategies after penetrating abdominal trauma at a level I urban trauma center: a comparison with the East guidelines. J Trauma.

[13] Smith BP, Fox N, Fakhro A, LaChant M, Pathak AS, Ross SE, Seamon MJ (2012). "SCIP"ping antibiotic prophylaxis guidelines in trauma: the consequences of noncompliance. J Trauma Acute Care Surg.

[14] Goldberg SR, Henning J, Wolfe LG, Duane TM (2017). Practice patterns for the use of antibiotic agents in damage control laparotomy and its impact on outcomes. Surg Infect (Larchmt).

[15] Herrod PJ, Boyd-Carson H, Doleman B (2019). Prophylactic antibiotics for penetrating abdominal trauma: duration of use and antibiotic choice. Cochrane Database Syst Rev.

[16] Mazuski JE, Symons WJ, Jarman S (2023). Reduction of surgical site infection after trauma laparotomy through use of a specific protocol for antibiotic prophylaxis. Surg Infect (Larchmt).

[17] Brand M, Grieve A (2013). Prophylactic antibiotics for penetrating abdominal trauma. Cochrane Database Syst Rev.

[18] Rosenberger LH, Politano AD, Sawyer RG (2011). The surgical care improvement project and prevention of post-operative infection, including surgical site infection. Surg Infect (Larchmt).

[19] de Jonge SW, Gans SL, Atema JJ, Solomkin JS, Dellinger PE, Boermeester MA (2017). Timing of preoperative antibiotic prophylaxis in 54,552 patients and the risk of surgical site infection: a systematic review and meta-analysis. Medicine (Baltimore).

[20] Itani KM, Wilson SE, Awad SS, Jensen EH, Finn TS, Abramson MA (2006). Ertapenem versus cefotetan prophylaxis in elective colorectal surgery. N Engl J Med.

[21] Hostler CJ, Krishnan J, Parish A (2024). Postoperative outcomes after receipt of ertapenem antimicrobial prophylaxis for colon surgery: a multicenter retrospective cohort study. Infect Control Hosp Epidemiol.

[22] Durbin S, DeAngelis R, Peschman J, Milia D, Carver T, Dodgion C (2019). Superficial surgical infections in operative abdominal trauma patients: a trauma quality improvement database analysis. J Surg Res.

[23] Wei S, Green C, Kao LS, Padilla-Jones BB, Truong VT, Wade CE, Harvin JA (2019). Accurate risk stratification for development of organ/space surgical site infections after emergent trauma laparotomy. J Trauma Acute Care Surg.

[24] Saccomanno FR, Gates J, Jacobs L, Kuti J, Ricaurte D, Keating J (2022). Infection and antibiotic agents in bleeding trauma patients: a review of available literature. Surg Infect (Larchmt).

[25] Ericsson CD, Fischer RP, Rowlands BJ, Hunt C, Miller-Crotchett P, Reed L 2nd (1989). Prophylactic antibiotics in trauma: the hazards of underdosing. J Trauma.

[26] Dellinger EP (2016). Teamwork and collaboration for prevention of surgical site infections. Surg Infect (Larchmt).

